# Mce transport systems in *Mycobacterium tuberculosis*: architecture, regulation, lipid import, and translational perspectives

**DOI:** 10.1128/jb.00221-26

**Published:** 2026-07-27

**Authors:** Haishan Tan, Yuxiang Hu, Qinglan Wang

**Affiliations:** 1 Institute of Respiratory Health, Frontiers Science Center for Disease-related Molecular Network, West China Hospital, Sichuan University, Chengdu, China; University of Notre Dame, Notre Dame, Indiana, USA

**Keywords:** *Mycobacterium tuberculosis*, Mce system, lipid transport, regulation, virulence, ABC transporter

## Abstract

Mammalian cell entry (Mce) systems are now recognized as central determinants of lipid uptake, cell-envelope homeostasis, and host adaptation in *Mycobacterium tuberculosis* (Mtb). Although the term “mammalian cell entry” originated from early studies linking the Mce loci to host-cell invasion phenotypes, subsequent genetic, biochemical, and structural work has substantially reframed their biological significance. Current evidence supports a model in which Mce1 and Mce4 function as multiprotein lipid-import systems specialized primarily for fatty-acid and cholesterol uptake, respectively, whereas shared and accessory factors, including MceG, LucA, Mam/Omam proteins, and the negative regulator Mce1N, govern transporter assembly, stability, and activity. At the same time, several individual Mce proteins have been implicated in host signaling and immunomodulation, although the physiological relevance of these observations remains unevenly established. In this review, we synthesize current knowledge of Mtb Mce systems with an emphasis on transporter organization, regulatory mechanisms, functional specialization, and biological significance during infection. We further discuss evolutionary relationships with other Mce-domain proteins, assess the strength of evidence supporting reported host-interaction phenotypes, and evaluate the translational potential of Mce biology for diagnostics, vaccines, and drug discovery. By emphasizing recent structural and regulatory breakthroughs, this review repositions Mce biology from a historically entry-centered narrative toward a transporter-centered, evidence-graded framework and highlights the key unresolved questions that should guide the next phase of the field, including substrate-level specificity, native structural validation, and the mechanistic integration of lipid transport with metabolism and virulence.

## INTRODUCTION

Tuberculosis remains one of the world’s leading causes of death from a single infectious agent. According to the World Health Organization (1–3), an estimated 10.7 million people developed tuberculosis in 2024, underscoring the continued importance of understanding the physiological mechanisms that enable *Mycobacterium tuberculosis* (Mtb) to persist within the host. Among the most distinctive features of Mtb pathogenesis is its reliance on host-derived lipids and its capacity to maintain a highly specialized, lipid-rich cell envelope that simultaneously supports nutrient acquisition, stress resistance, and immune modulation. Against this backdrop, lipid transport systems have emerged as core determinants of intracellular adaptation in Mtb.

The mammalian cell entry (Mce) loci were originally identified through phenotypes linked to bacterial entry into mammalian cells, giving rise to terminology that has persisted in the field for decades (4). However, this historical framing is no longer sufficient. Accumulating evidence now indicates that mycobacterial Mce proteins are best understood primarily as components of multiprotein lipid transport systems rather than as simple invasion factors. In Mtb, four Mce operons, designated Mce1 through Mce4, encode related but functionally distinct transport modules that contribute to lipid uptake, envelope homeostasis, and host adaptation. Among these, Mce1 and Mce4 are currently the best characterized, with compelling evidence linking them to fatty-acid and cholesterol uptake, respectively (5). Accordingly, any contemporary review of Mce proteins should treat the “entry factor” concept as historically important but mechanistically incomplete.

Recent years have brought substantial conceptual advances to the field. Cryo-electron microscopy has revealed the architecture of an endogenous mycobacterial Mce1 transporter, providing a structural framework for understanding how MceA–F subunits, YrbE permeases, and associated factors assemble into an elongated lipid-import machine (6). In parallel, regulatory studies have identified Mce1N as a negative regulator that interferes with productive transporter assembly, while genetic and proteomic analyses have further clarified the roles of LucA and MceG in stabilizing and energizing Mce complexes (7–10). Together, these findings have shifted the field from a largely phenotype-driven literature toward a more mechanistic understanding of Mce biology and make it timely to revisit the field from a transporter-centered perspective.

**Fig 1:**
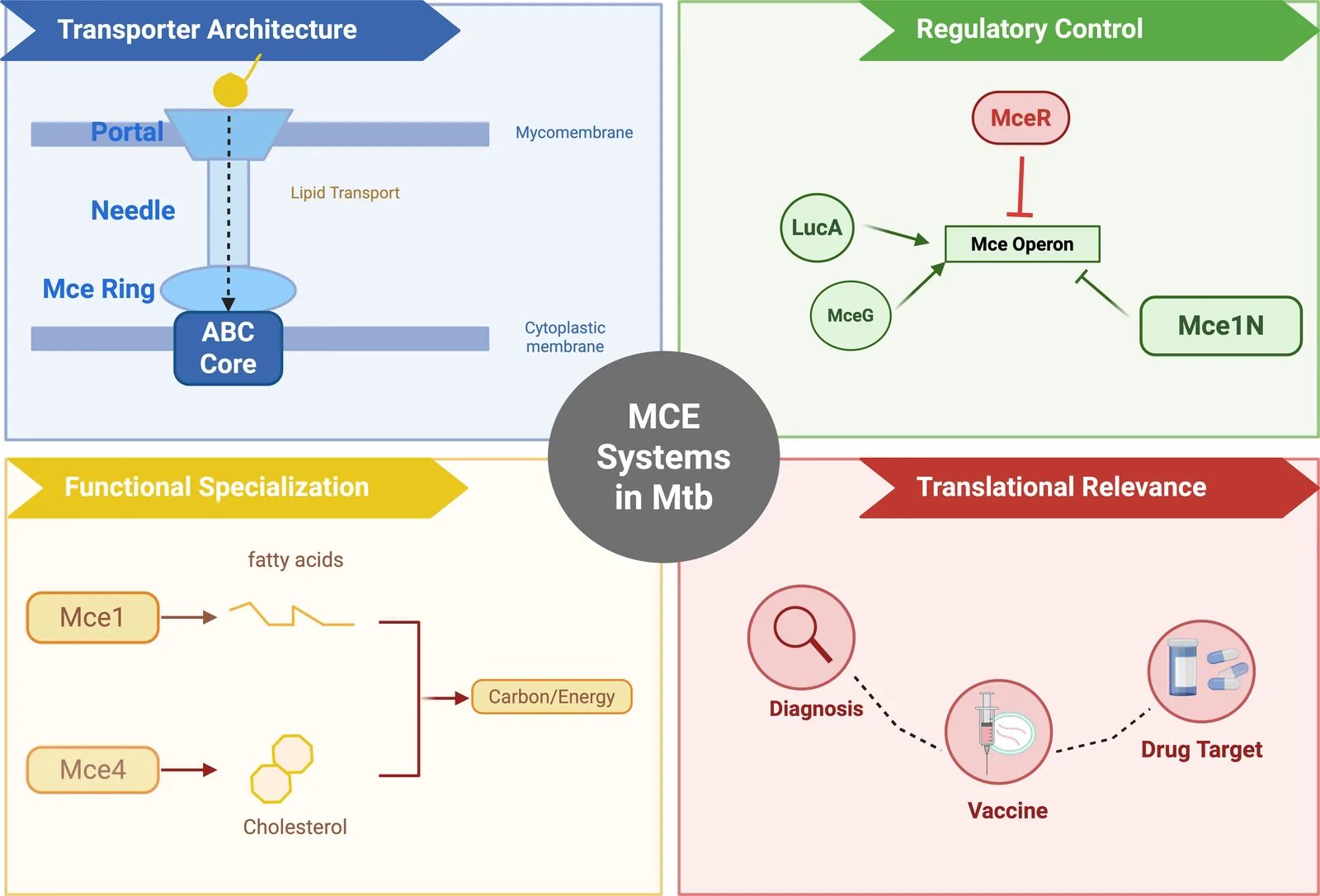
Schematic overview of Mce systems in *Mycobacterium tuberculosis*. Transporter architecture (blue): the Mce complex is depicted as a trans-envelope lipid conduit comprising a portal region, an elongated tunnel-like assembly, an Mce-domain ring, and an ATP-driven inner-membrane core. Regulatory control (green): Mce activity is modulated by transcriptional regulators, by stabilizing factors such as LucA and MceG, and by the negative post-translational regulator Mce1N. Functional specialization (yellow): current evidence most strongly supports a major role for Mce1 in fatty-acid uptake and for Mce4 in cholesterol uptake. Translational relevance (red): Mce systems provide candidate nodes for diagnostics, vaccines, and drug discovery. This schematic emphasizes the best-supported features of the Mce1/Mce4 framework; several aspects of stoichiometry, operon-specific architecture, and substrate trajectory remain to be refined for other Mce systems.

Despite this progress, major questions remain unresolved. Not all Mce operons are equally well understood; the physiological substrate range of several systems remains unclear, and the interpretation of host-interaction phenotypes reported for individual Mce proteins often exceeds the strength of direct mechanistic evidence. In this review, we examine Mce systems in Mtb from four interrelated perspectives: transporter architecture, regulatory control, functional specialization in lipid uptake and envelope biology, and translational relevance (Fig. 1). We also discuss how comparative and evolutionary analyses can inform, but should not overdetermine, the interpretation of mycobacterial Mce function.

A key aim of this review is therefore not merely to summarize prior literature, but to highlight what is new in the field: the emergence of a transporter-centered structural framework, the identification of post-translational regulatory logic, and the need to separate well-supported transporter biology from more heterogeneous reports of host-modulatory activity.

## GENETIC ORGANIZATION AND GENERAL ARCHITECTURE OF Mce SYSTEMS

### Conserved operon organization

The genome of Mtb encodes four paralogous Mce operons (Mce1–Mce4), each with a highly conserved core architecture characteristic of actinobacterial Mce systems (11). Each operon contains a canonical eight-gene module consisting of two yrbE genes (yrbEA, yrbEB) and six Mce genes (MceA–MceF), which together encode the major structural subunits of a lipid-import complex. This overall organization is broadly conserved across mycobacteria and related actinobacterial Mce systems (12, 13).

The YrbE proteins function as inner-membrane permease subunits homologous to ABC transporter permeases, typically containing five transmembrane helices and sequence features implicated in interaction with the ATPase subunit (14). The six Mce proteins (MceA–F) each harbor a defining Mce domain, a ~100-amino-acid β-barrel fold involved in lipid handling and complex assembly. Some Mce proteins also contain short sequence motifs, including RGD-like segments, that have been implicated in host interaction in specific experimental settings; however, the extent to which such motifs represent a general family-wide feature and their physiological relevance during native infection remain unclear.

Together, the YrbE permeases and Mce substrate-binding proteins assemble into an ABC-transporter-like machinery that spans the mycobacterial cell envelope (6). The periplasmic MceA–F heterohexamer interacts with the inner-membrane YrbEA/B heterodimer, which in turn associates with the shared ATPase MceG to drive substrate translocation via ATP hydrolysis. Beyond the eight-gene core, each operon includes a distinct set of downstream accessory genes, most notably mam genes, which contribute to complex stability and function but are not uniformly conserved across all four operons.

Notably, while the core operon structure is conserved, each Mce cluster has undergone diversification in accessory genes and regulatory inputs that likely contributes to functional specialization during infection. This combination of a shared structural framework with variable accessory and regulatory features may help explain how the four systems can support uptake of distinct host lipids while retaining common mechanistic principles.

### Structural framework of the Mce1 transporter

The near-atomic cryo-EM structure of the endogenous mycobacterial Mce1 complex (2.3–3.2 Å) has defined its architectural framework, revealing an elongated, trans-envelope lipid-import machine adapted to the thick mycobacterial cell envelope (6). Determined in *Mycobacterium smegmatis* as a model for mycobacterial lipid transport, this structure provided the first experimentally grounded architecture for a mycobacterial Mce system and substantially advanced the mechanistic interpretation of lipid translocation across the mycolic acid-rich envelope.

The Mce1 complex is an approximately 310 Å-long lipid transporter assembled from 10 distinct subunits, including one copy each of YrbE1A, YrbE1B, and Mce1A–F, together with two copies of the ATPase MceG and the accessory subunit LucB (Rv2536) (6). Structurally, the complex is organized into four interconnected modular regions that together create a continuous hydrophobic pathway for fatty-acid transport across the mycobacterial envelope. At the outer membrane, the portal domain is formed by the C-terminal β-barrel regions of Mce1A–F and likely serves as the initial lipid-entry site for fatty acids and mycolic acids. Extending inward from the portal is a long, curved, needle-like assembly of approximately 225 Å, generated by stacked α-helical repeats from the Mce1A–F subunits, which together form a sealed hydrophobic tunnel that protects lipid substrates during transit through the hydrophilic periplasmic space. Beneath this structure lies the heterohexameric Mce ring, composed of the six Mce domains, which functions as a gating interface controlling substrate passage toward the inner-membrane transporter. Finally, the ABC core consists of the YrbE1A/YrbE1B permease heterodimer and an MceG ATPase homodimer, which couples ATP hydrolysis to substrate translocation across the inner membrane.

Notably, Mce1 assembles as a heterohexamer (Mce1A–F), in contrast to the homohexameric Mce complexes of gram-negative bacteria (e.g., MlaD) (15). LucB is a Mce1-specific four-transmembrane subunit that binds Mce1C and stabilizes the complex, with no homolog in *Escherichia coli* or other Mce systems. Biochemical studies further show that MSMEG_6540 (an Mce1A paralog) can replace Mce1A in the complex, generating functionally distinct Mce1 assemblies with differential substrate preference (7).

This structural framework establishes Mce1 as a specialized ABC-type lipid importer with a distinctive needle-and-portal architecture adapted for long-chain hydrophobic lipid uptake. At present, however, this architectural framework is most firmly established for the Mce1 system. Whether equivalent stoichiometric and conformational principles extend fully to the Mce2 and Mce3 systems remains unresolved and should not be assumed.

### Accessory proteins and system complexity

Beyond the core YrbEAB permeases and MceA–F substrate-binding subunits, mycobacterial Mce systems rely on a suite of accessory proteins that likely influence complex assembly, structural stability, and functional tuning. These factors expand the biological complexity of Mce machineries and help adapt lipid import to the demands of the host intracellular niche.

LucB (Rv2536) is a Mce1-specific tetraspan accessory subunit identified via cryo-EM and mass spectrometry (6). It inserts into the inner membrane and binds directly to the transmembrane domain of Mce1C via a conserved cleft formed by its TM2 and TM4 helices, stabilizing the Mce1 complex and modulating transport activity. LucB is absent from Mce4 and has no homologs in gram-negative bacteria, representing an actinomycete-specific innovation for hetero-oligomeric Mce regulation.

Mam (Mce-associated membrane) proteins are encoded downstream of most Mce operons and contribute to complex stability and function (16). The Mce 1, Mce 3, and Mce 4 operons carry four, two, and two mam genes, respectively, whereas no mam genes flank Mce 2. Mam4A and Mam4B are important components of the Mce4 complex and are required for full structural integrity and function during infection (17).

Omam (orphan Mce-associated membrane) proteins are Mam-family members encoded outside Mce operons. OmamA (Rv0199) and OmamB (Rv0200) stabilize both Mce1 and Mce4, thereby supporting fatty-acid and cholesterol import and contributing to full virulence (16, 18). OmamA contains an N-terminal cytoplasmic region, a single transmembrane helix, and a C-terminal NTF2-like fold that extends toward the cell envelope and is thought to mediate interactions with Mce complexes. While roles for OmamA and OmamB are established, the functions of OmamC–F remain largely unresolved (13).

## REGULATORY LOGIC OF Mce TRANSPORTERS

### MceG as a shared ATPase

MceG (Rv0655) serves as the conserved shared ATPase for multiple Mce transport systems (Mce 1–4) in Mtb. It functions as the energy-coupling component associated with YrbEAB inner-membrane permeases and is required for productive lipid transport. Recent work further indicates that MceG contributes to the stability of the Mce1 and Mce4 transporters, linking ATPase recruitment not only to energy supply but also to maintenance of transporter integrity (9, 17, 18). MceG contains a canonical ATPase domain with Walker A and Walker B motifs, and mutational analysis has shown that these motifs are required for normal transporter function and/or protein stability (10). Collectively, current evidence supports a dual role for MceG in both energy coupling and maintenance of transporter integrity, particularly for the Mce1 and Mce4 systems.

### LucA and stabilization of lipid uptake pathways

LucA (Rv3723) functions as a coordinator of lipid uptake pathways that stabilizes both Mce1 and Mce4 in Mtb, rather than acting as a direct transport pump. Forward genetic screening first identified LucA as essential for growth on cholesterol as the sole carbon source. Quantitative assays using ^14^C-cholesterol and Bodipy-C^16^ fatty-acid probes confirmed that ΔlucA strains exhibit markedly reduced cholesterol uptake together with profound defects in fatty-acid acquisition in macrophages (8). Transcriptomic analysis further showed altered expression of lipid metabolic regulons in the deletion mutant, linking LucA to broader metabolic adaptation. LucA appears to act as a molecular scaffold that interacts with Mce1 and Mce4 complexes and helps prevent their degradation, thereby maintaining structural integrity and transport activity. In mouse infection models, LucA deficiency markedly attenuates persistence and virulence, underscoring its importance for host adaptation. However, the precise molecular interfaces through which LucA engages the Mce1 and Mce4 machineries remain incompletely defined (18).

### Mce1N as a post-translational negative regulator

Mce1N (MSMEG_0959 in *Mycobacterium smegmatis* and Rv0513 in Mtb) is a post-translational negative regulator that specifically inhibits the Mce1 fatty-acid import system through an assembly-blocking mechanism. Co-immunoprecipitation and mass spectrometry identified Mce1N as a membrane protein associated with core Mce1 components. Mechanistically, Mce1N directly binds to the YrbE1B permease subunit, and this binding site sterically clashes with the recruitment of the shared ATPase MceG (7).

This mutually exclusive interaction competitively blocks assembly of a productive Mce1 transporter and thereby inhibits fatty-acid uptake. Structural analysis of the Mce1N-associated complex clarified the basis of this inhibitory interaction and established Mce1N as one of the clearest examples of post-translational control in the Mce field. The Mtb homolog Rv0513 appears to retain the same repressive logic.

### Transcriptional regulation of Mce loci

MceR-family transcriptional regulators tightly govern the expression of Mce operons in Mtb, coupling lipid uptake to host environment, nutrient availability, and stress responses (13). Each Mce locus is controlled by distinct repressors, resulting in non-redundant, infection-stage-specific expression patterns. The transcriptional regulators, KstR1 and Mce3R, have been classified as TetR-type regulators (19–24), while Mce1R and Mce2R belong to VanR and FadR types of regulators, respectively (25–29).

Multiple transcriptional regulators govern Mce operon expression in Mtb, linking lipid uptake to nutrient availability, environmental cues, and stress adaptation (30). These regulators include both locus-associated repressors and more distant metabolic regulators, likely contributing to non-redundant and context-dependent expression patterns across the four operons.

Mce1R is a fatty-acid-responsive repressor that has been linked to control of the Mce operon. The binding of long-chain fatty acids has been proposed to weaken promoter occupancy, thereby favoring derepression of Mce1 expression and enhanced fatty-acid uptake under intracellular conditions (31). Mce2R represses the Mce2 operon and has also been linked to the regulation of stress-associated genes, suggesting a broader interface between lipid-related regulation and survival under hostile intracellular conditions (27). Mce3R is an atypical TetR-type regulator that adopts an asymmetric architecture and binds non-palindromic operator sequences. Recent work suggests that Mce3R helps coordinate responses to lipid availability and environmental stress, thereby linking the Mce3 region to intracellular adaptation rather than providing direct proof of a defined transport substrate for the Mce3 machinery (20, 22).

In contrast, the Mce4 operon is not controlled by a local MceR-type repressor but instead falls under the broader cholesterol-responsive KstR regulatory network (23, 24). This distinction reinforces the view that transcriptional control of Mce loci is embedded within larger metabolic circuits rather than governed by a single uniform logic.

## FUNCTIONAL SPECIALIZATION OF Mce SYSTEMS AND CELL-ENVELOPE HOMEOSTASIS

### Mce1-mediated fatty-acid uptake and envelope maintenance

The Mce1 system is currently the best-supported fatty-acid uptake transporter in Mtb and contributes importantly to intracellular proliferation, cell-envelope homeostasis, and virulence (32–34). It mediates uptake of host-derived long-chain fatty acids across the complex mycobacterial envelope, thereby supporting membrane biogenesis, energy production, and metabolic adaptation within macrophages. Deletion of the Mce1 operon impairs uptake of palmitic acid and related long-chain fatty acids, leading to abnormal free mycolic-acid accumulation, altered lipid composition, and disruption of envelope homeostasis. These defects compromise bacterial fitness in macrophages and in mouse lungs. Cryo-EM analysis further revealed that the Mce1 complex forms an elongated trans-envelope transporter containing a continuous hydrophobic tunnel that plausibly shields lipid substrates during transit (6).

Beyond direct fatty-acid uptake, Mce1 activity appears closely linked to broader envelope physiology. The altered lipid profiles and membrane-associated phenotypes observed in Δmce1 strains suggest that Mce1-mediated transport influences not only nutrient acquisition but also maintenance of proper cell-wall organization (35). Although the precise mechanistic connection between fatty-acid transport and envelope remodeling remains incompletely resolved, current evidence strongly supports a central role for Mce1 in maintaining envelope integrity during infection (36). Regulatory proteins further fine-tune Mce1 activity. LucA stabilizes the transporter complex to ensure efficient lipid uptake, whereas Mce1N acts as a post-translational negative regulator that likely prevents excessive lipid influx and associated metabolic imbalance during persistence. Despite growing mechanistic insight, the full substrate range and recognition logic of the Mce1 system remain incompletely understood.

### Mce4-mediated cholesterol uptake and metabolic adaptation

The Mce4 system is the best-supported cholesterol importer in Mtb and is essential for efficient cholesterol utilization and long-term persistence during infection (5). Early genetic studies demonstrated that the Mce4 operon is required for growth on cholesterol as a sole carbon source, establishing one of the clearest functional links between an Mce system and a defined host-derived lipid. Subsequent genetic and biochemical studies further confirmed that multiple Mce4-associated proteins contribute to cholesterol uptake and intracellular survival. Structural and biochemical analyses of purified Mce4A are also consistent with cholesterol-binding activity, although the detailed transport mechanism of the intact Mce4 complex remains unresolved (37–39).

Like Mce1, Mce4-dependent lipid uptake also appears closely integrated with broader envelope and metabolic physiology. Cholesterol catabolism contributes propionyl-CoA and other intermediates to downstream lipid metabolism, suggesting that Mce4 activity may indirectly influence cell-envelope composition and stress adaptation (40, 41). Changes in Mce4 expression under hypoxic or membrane-stress conditions further support the view that cholesterol uptake is functionally coupled to metabolic remodeling and envelope homeostasis (42). Although ΔMce4 mutants may retain limited cholesterol utilization through auxiliary uptake routes, Mce4 remains the dominant cholesterol-import pathway during infection (5). Iron availability additionally influences downstream sterol catabolism, indicating that the physiological consequences of Mce4-dependent uptake vary according to the broader metabolic state of the bacterium (43).

### Functional asymmetry and pleiotropic phenotypes across the four operons

Despite shared structural organization, the four Mce operons are not functionally equivalent. Genetic interaction studies support both distinct physiological specialization and limited crosstalk among the pathways. Simultaneous disruption of Mce1 and Mce4 produces more severe infection defects than deletion of either system alone, indicating partial functional overlap that nevertheless remains important for *in vivo* survival. By contrast, the functions of Mce2 and Mce3 remain considerably less defined. Disruption of Mce2 has been associated with altered sulfolipid accumulation, although it remains unclear whether Mce2 directly mediates sulfolipid transport or instead affects lipid homeostasis indirectly (44, 45). Likewise, the substrate spectrum and primary physiological role of the Mce3 system remain incompletely characterized.

Disruption of Mce systems also produces pleiotropic morphological and physiological phenotypes consistent with impaired envelope integrity. In *Mycobacterium avium* subsp. *paratuberculosis*, deletion of the Mce4 operon causes rough colony morphology and increased cellular aggregation, phenotypes suggestive of cell-wall structural abnormalities (46). At present, the mechanistic basis of these phenotypes remains unresolved. They may arise from insufficient lipid precursors for envelope biosynthesis, secondary metabolic consequences of impaired transport, defective assembly of Mce complexes themselves, or combinations of these processes. Resolving these possibilities will be important for understanding how Mce-mediated lipid transport interfaces with broader mycobacterial envelope physiology.

## Mce SYSTEMS IN VIRULENCE AND HOST ADAPTATION

### Intracellular survival and persistence

Mce systems make important contributions to intracellular survival, long-term persistence, and virulence by integrating host lipid acquisition, cell-envelope homeostasis, and adaptation to hostile intracellular environments.

Mce1 contributes to intracellular fitness by supporting host long-chain fatty-acid uptake and envelope homeostasis. Deletion of the Mce1 operon impairs lipid acquisition, perturbs cell-wall lipid composition, and reduces bacterial fitness in macrophages and mouse lungs (35, 47, 48). Regulation at both transcriptional and post-translational levels, including inputs from Mce1R and Mce1N, likely helps match fatty-acid uptake to intracellular demand (49).

Mce4 is central to cholesterol utilization and is particularly important during chronic infection. Δmce4 mutants are impaired in cholesterol-dependent growth and show reduced survival in activated macrophages and during later phases of infection (50). LucA coordinates both Mce1-linked and Mce4-linked uptake pathways, and ΔlucA strains display severe defects in both cholesterol and fatty-acid acquisition together with marked attenuation *in vivo* (8).

System-level factors further reinforce the contribution of Mce biology to persistence. MceG is required for the stable functioning of multiple Mce transporters, and accessory proteins such as Mam4A/B and OmamA/B support the integrity of the Mce1 and Mce4 machineries (16). These observations point to virulence phenotypes that emerge not only from loss of a specific substrate route but also from destabilization of the transport system as a whole.

Some studies also suggest that individual Mce proteins may influence persistence through host-modulatory activities (51, 52). However, the strength of evidence for these effects is heterogeneous and should be distinguished from the more direct transporter-centered evidence described above. Taken together, current evidence indicates that Mce systems make important contributions to persistence by coupling host lipid acquisition, envelope maintenance, and adaptation to intracellular stress.

### Host interaction and immunomodulation by individual Mce proteins

**Fig 2:**
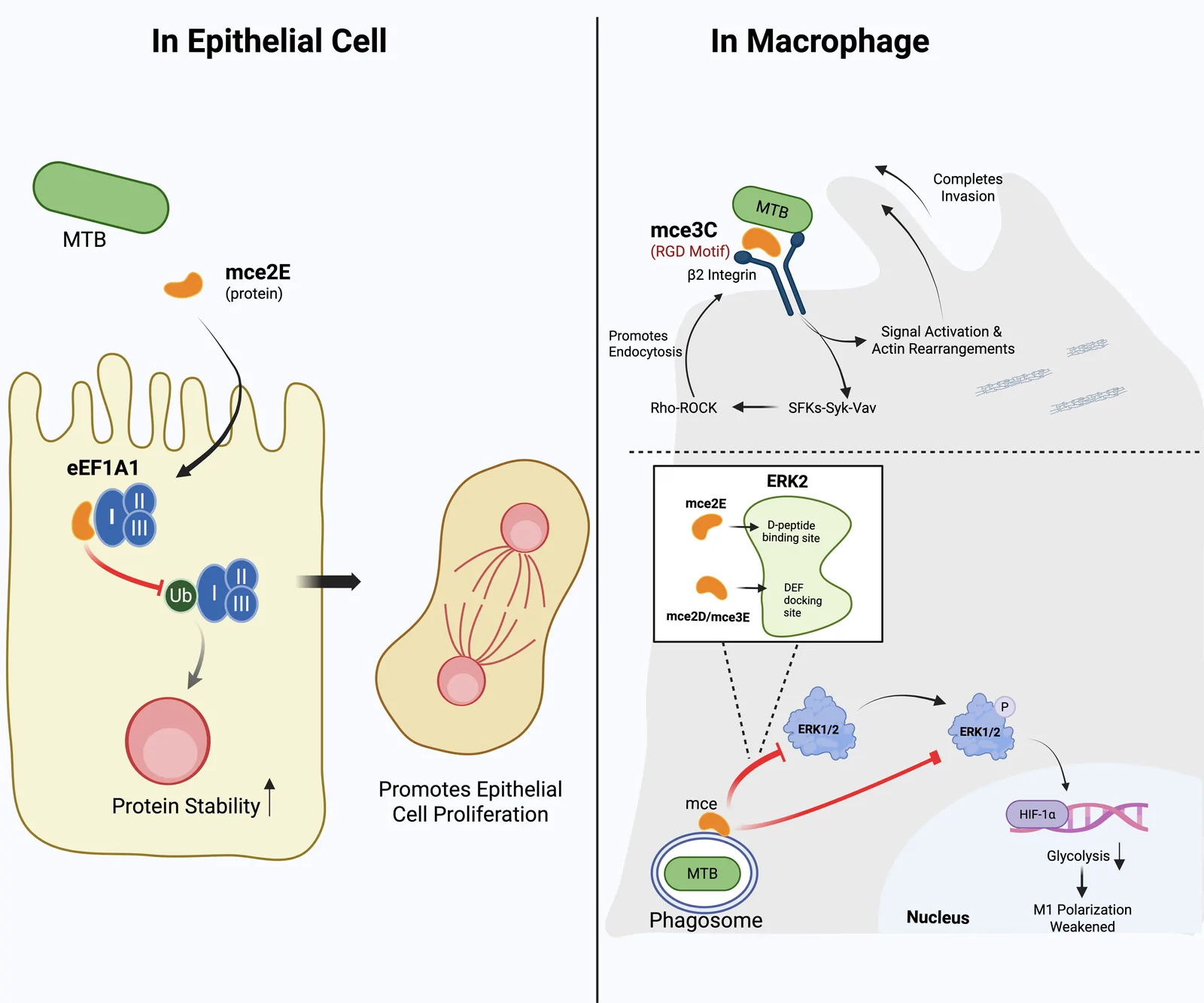
Reported host-cell interaction models for selected Mce proteins. This figure summarizes assay-based models in which selected Mce proteins have been linked to host signaling or cellular phenotypes. In epithelial-cell systems, Mce2E has been reported to interact with eEF1A1 and to influence protein stability and proliferation. In macrophage-focused assays, Mce3C has been linked to β2-integrin-associated signaling and bacterial entry, whereas Mce2D, Mce2E, and Mce3E have been reported to affect ERK-related signaling and inflammatory output. These models are informative but derive from heterogeneous experimental systems and therefore should not be interpreted as equivalent levels of physiological evidence.

Some individual Mce proteins have been proposed to exert host-modulatory or “moonlighting” activities beyond their roles within intact lipid-transport complexes (Fig. 2). Reported activities include effects on host signaling pathways, inflammatory output, macrophage polarization, and epithelial-cell proliferation. However, these observations were generated in heterogeneous experimental systems and should be interpreted cautiously unless supported by native localization, loss-of-function genetics, and infection-context validation.

Evidence for host interaction by individual Mce proteins derives from heterogeneous experimental systems, including recombinant protein stimulation, heterologous or ectopic expression, infection with mutant strains, and pathway-focused cell biology assays (53–55). These studies are informative and often mechanistically interesting, but they do not all provide the same level of physiological evidence. Accordingly, reported host-modulatory activities should be interpreted separately from the transporter-centered functions of intact Mce complexes.

Mce3E has been reported to suppress innate immune signaling by targeting the ERK1/2 pathway in macrophage-based assays, thereby reducing production of TNF-α and IL-6 and promoting a more permissive intracellular environment (56).

Mce2E has been reported to exert cell-type-dependent effects. In macrophages, it can dampen MAPK signaling and pro-inflammatory cytokine production, whereas in epithelial-cell systems, it has been linked to stabilization of eEF1A1 and enhanced cell proliferation (54).

Mce2D has likewise been reported to inhibit ERK-associated signaling and to reduce macrophage M1 polarization in cellular assays (57). Together, these observations suggest that some Mce proteins may converge on host signaling axes relevant to innate immune control, although the extent to which this occurs under native infection conditions remains uncertain.

Mce3C has been implicated in host-cell entry and immune evasion through β2 integrin-dependent signaling in macrophage assays (55). These findings are interesting, but whether such interactions represent a broadly deployed physiological function of surface-exposed Mce proteins during infection remains to be clarified.

More generally, sequence motifs such as RGD-like segments have prompted the hypothesis that selected Mce proteins may interact with host receptors (58). At present, however, such motif-based interpretations should be treated cautiously unless supported by native localization, loss-of-function, and infection-context validation. Collectively, available studies suggest that some individual Mce proteins may possess host-modulatory activities beyond canonical lipid transport. However, the extent to which these observations reflect native protein abundance, localization, and *in vivo* relevance remains incompletely resolved.

## EVOLUTIONARY AND COMPARATIVE PERSPECTIVES

### Mce-domain proteins across biological systems

**Fig 3:**
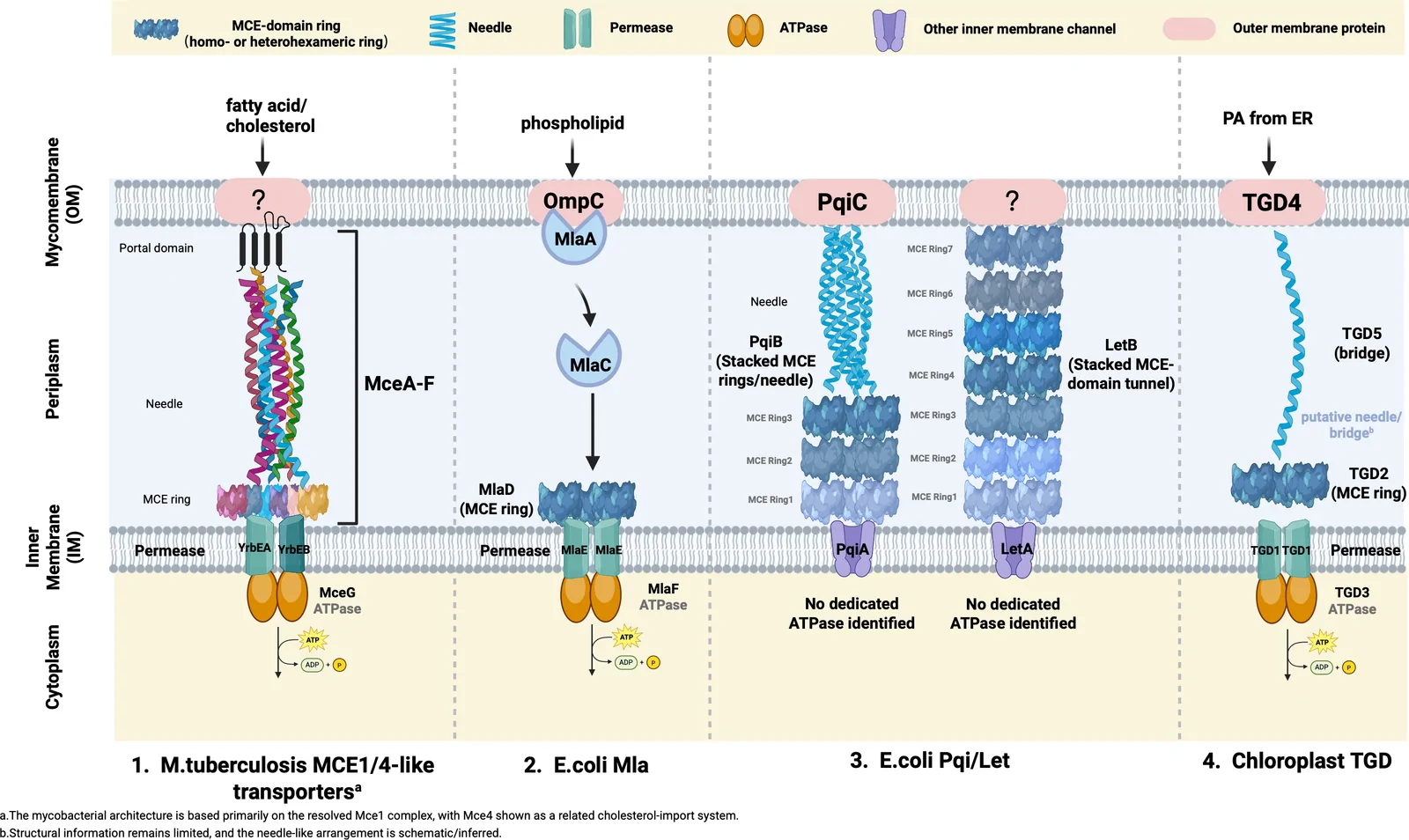
Comparative schematic of representative Mce-domain transport systems. The diagram compares mycobacterial Mce1/4-like transporters, the *E. coli* Mla pathway, *E. coli* Pqi/Let systems, and the chloroplast TGD complex. Similar structural elements are depicted using consistent graphical symbols, including Mce-domain rings, elongated needle-like or tunnel-like assemblies, inner-membrane permeases or channels, ATPases, and outer-membrane-associated proteins. The mycobacterial model is based primarily on the structurally resolved Mce1 transporter and illustrates a heterohexameric MceA–F assembly associated with YrbE permeases, MceG ATPase, and accessory factors. In contrast, Mla, PqiB, LetB, and TGD2-related systems are shown as homo-oligomeric or predicted homo-oligomeric Mce-domain assemblies adapted to distinct envelope contexts. For Pqi, Let, and TGD systems, several aspects of substrate trajectory, transport directionality, and full native architecture remain incompletely resolved; therefore, these models are intended to highlight conserved architectural principles rather than imply complete mechanistic equivalence.

Mce domains represent an ancient, structurally conserved lipid-binding module found across diverse diderm bacteria and chloroplast-related systems, where they are broadly associated with membrane lipid trafficking and envelope homeostasis (Fig. 3). Comparative analysis is useful because it clarifies which properties of Mce systems are likely ancient and shared, and which appear to be lineage-specific innovations in mycobacteria (Table 1).

**TABLE 1:** Comparative overview of representative Mce-domain systems across species, including gram-negative Mla/Pqi/Let pathways, TGD-like systems, and mycobacterial Mce systems^*a*^

Feature	Mla system (maintenance of lipid asymmetry)	Pqi system (paraquat-inducible/ envelope stress-associated)	Let system (lipid envelope transporter)	TGD-like system (phosphatidic acid transporter)	Mycobacterial Mce system (cholesterol/fatty-acid transporter)
Core function	Retrograde phospholipid transport from the outer membrane toward the inner membrane to maintain outer-membrane lipid asymmetry.	Envelope lipid homeostasis; induced by envelope/oxidative stress and implicated in transport or clearance of aberrant lipids.	Stress-responsive trans-envelope lipid transport that contributes to envelope homeostasis, particularly when outer-membrane integrity is compromised.	Transport of endoplasmic reticulum-derived phosphatidic acid across the chloroplast envelope for galactolipid biosynthesis.	Host-lipid acquisition and mycomembrane/envelope remodeling; best-supported examples include Mce1-mediated fatty-acid uptake and Mce4-mediated cholesterol uptake.
Core Mce-domain protein	MlaD	PqiB	LetB (YebT)	TGD2	MceA–F family proteins
Number of Mce domains	One Mce domain per MlaD subunit.	Three tandem Mce domains in PqiB.	Seven tandem Mce domains in LetB.	One Mce domain in TGD2.	One Mce domain per MceA–F subunit; six different subunits assemble into a heterohexameric complex.
Assembly architecture	Homohexameric MlaD ring associated with the MlaFEDB inner-membrane ABC transporter and a soluble carrier, MlaC.	PqiB forms an extended periplasmic Mce-domain assembly associated with PqiA and PqiC.	LetB forms a large hexameric tunnel-like assembly spanning the periplasm.	TGD components form a chloroplast-envelope lipid-transport system with TGD2 as the Mce-domain component and TGD5 as a bridging factor.	Heterohexameric MceA–F ring plus extended helical needle/tunnel, YrbEA/B inner-membrane permeases, MceG ATPase, and accessory factors such as LucA/LucB and Mam/Omam proteins.
System components	MlaA (OM), MlaC (periplasm), MlaFEDB (IM).	PqiA (IM), PqiB (periplasmic Mce protein), PqiC (OM lipoprotein).	LetA (IM; also known as YebS), LetB (periplasmic Mce tunnel protein).	TGD1 (IM permease), TGD2 (intermembrane/peripheral Mce-domain protein), TGD3 (ATPase), TGD4 (OM), TGD5 (bridging component).	YrbEA/YrbEB (IM permeases), MceA–F (envelope-spanning substrate-binding/transport subunits), MceG (ATPase), plus operon-specific accessory proteins.
Expression/regulation	Often described as a housekeeping envelope-maintenance pathway.	Inducible under stress conditions; regulation has been linked to envelope and oxidative-stress responses.	Inducible under membrane stress or specific growth conditions.	Metabolically regulated in the context of chloroplast lipid biosynthesis.	Regulated by locus-specific and metabolic regulators; expression and activity are tuned during infection, host-lipid utilization, and envelope stress.

^*a*^ OM, outer membrane; IM, inner membrane.

In gram-negative bacteria, several Mce-related systems, including Mla, Pqi, and Let, have been identified and shown to possess distinct architectures, transport mechanisms, and physiological functions (11, 59–62). Mce-domain proteins assemble into diverse lipid transport complexes through oligomerization and combination with additional structural domains, thereby mediating lipid trafficking across the bacterial envelope.

Among these systems, the Mla pathway has been studied most extensively. MlaD contains a single Mce domain and assembles into a hexameric ring with a central hydrophobic pore. Together with the transmembrane permease MlaE, the ATPase MlaF, and the accessory protein MlaB, it forms the inner-membrane MlaFEDB complex. Structural analyses revealed that MlaE forms a dimeric transmembrane domain with a hydrophobic substrate-binding cavity, whereas MlaF exhibits a canonical ABC transporter fold, and MlaB stabilizes the transporter complex (59, 60, 63, 64). Molecular dynamics simulations further suggested that the β6–β7 loop of MlaD plays an essential role in phospholipid translocation, with lipids passing through the C-terminal helices of the MlaD hexamer into the central channel. The mycobacterial Pqi system is proposed to function in envelope lipid homeostasis by mediating trans-envelope lipid transport, although its physiological substrates and transport directionality remain unresolved.

In addition to Mla, the PqiABC and LetAB systems have also been implicated in phospholipid transport. PqiABC consists of the inner-membrane protein PqiA, the periplasmic protein PqiB containing three tandem Mce domains, and the outer membrane lipoprotein PqiC (11, 12, 65). LetAB exhibits a related but structurally distinct organization. LetA is a six-transmembrane inner-membrane protein with topology similar to PqiA, whereas LetB is an unusually large Mce protein composed of seven tandem Mce domains. Six LetB monomers assemble into a stacked hexameric tunnel spanning the periplasm, potentially enabling direct lipid transport between the inner and outer membranes without the need for soluble carrier proteins (62). Notably, LetB may integrate the outer membrane anchoring function of PqiC through evolutionary gene fusion, allowing the formation of an autonomous trans-envelope lipid transport conduit.

Functionally, LetAB is thought to act as a stress-responsive lipid transport system that becomes important when outer membrane integrity is compromised, particularly under conditions of lipopolysaccharide deficiency. Under such circumstances, LetAB may function redundantly with the Mla and Pqi systems to preserve membrane homeostasis. However, the precise substrates, transport directionality, and regulatory coordination of the LetAB and PqiABC systems remain poorly understood. Whether these systems exhibit substrate specificity and how their functions are integrated within bacterial lipid homeostasis are still unresolved questions.

Mce-domain proteins are also present in plant chloroplasts. In chloroplasts, the trigalactosyldiacylglycerol (TGD) complex mediates the transport of endoplasmic reticulum-derived phosphatidic acid across the chloroplast envelope for galactolipid biosynthesis. The core transporter comprises the inner-membrane ABC transporter subunits TGD1, TGD2, and TGD3. TGD2 contains an Mce domain capable of specifically binding phosphatidic acid. In the outer membrane, TGD4 and TGD5 cooperate with the TGD1–2–3 complex, with TGD5 serving as a bridging component that links outer and inner-membrane subcomplexes (66–70). Together, these proteins mediate phosphatidic acid transport from the endoplasmic reticulum, across the chloroplast intermembrane space, and into the stroma-facing inner membrane.

In Mtb, Mce proteins possess a conserved four-domain organization consisting of a transmembrane domain, an Mce domain, a helical domain, and a C-terminal tail domain. The Mce domain adopts a β-barrel fold that forms a putative lipid transport channel, whereas the extended helical domains are proposed to mediate protein-protein interactions and stabilize complex assembly. Compared with homologous Mce proteins from gram-negative bacteria, such as MlaD, PqiB, and LetB, Mtb Mce proteins share the conserved β-barrel architecture but display low sequence identity (<15%), substantially longer helical domains, and distinct tail structures. Moreover, unlike the homohexameric assembly observed in MlaD, Mtb Mce complexes assemble into heterohexameric structures composed of different Mce subunits.

Across all of these systems, the Mce domain adopts a conserved β-barrel-like fold associated with hydrophobic substrate handling. However, divergence in oligomeric organization, accessory composition, and biological setting has produced considerable mechanistic diversity.

### Limits of mechanistic analogy and evolutionary interpretation

Although Mce-domain proteins share a conserved β-barrel fold across diderm bacteria and chloroplast systems, mechanistic extrapolation to Mtb is often unreliable. The most fundamental limitation arises from differences in complex assembly. In contrast to the homohexameric organization observed in gram-negative bacteria (e.g., MlaD, PqiB, and LetB) and chloroplast TGD components, mycobacterial Mce systems assemble as heterohexameric complexes (MceA–F). This heterogeneity is thought to enable substrate specialization, particularly for host-derived lipids, a feature not observed in canonical bacterial lipid homeostasis systems.

The distinctive architecture of the mycobacterial cell envelope, including a mycolic acid-rich outer layer, may influence the organization of Mce systems. Mce complexes are generally thought to function as elongated assemblies bridging the periplasmic space between the inner and outer membranes, rather than directly traversing the outer membrane. This bridging principle is also observed in gram-negative systems such as LetB and PqiB. In addition, related Mce-containing systems are found in plants (TGD systems) and gram-negative bacteria, where they are predicted to assemble into single Mce-ring structures with extended tunnel-like architectures and to associate with ABC transporters in the inner membrane (e.g., YrbE-like and MceG-like proteins). Nevertheless, detailed structural and mechanistic understanding of several of these systems remains incomplete.

In addition, Mtb encodes multiple lineage-specific accessory and regulatory factors, including MceG, LucA, LucB, Mam, Omam, and Mce1N, which lack clear orthologs in other Mce systems. These proteins modulate transporter assembly, activity, and substrate utilization, thereby adapting Mce function to intracellular survival, immune interaction, and long-term persistence rather than simple membrane lipid homeostasis.

From an evolutionary perspective, Mce domains likely originated as conserved lipid-binding modules involved in envelope maintenance in diderm bacteria. However, pathogenic mycobacteria have substantially expanded and rewired these systems through gene duplication and regulatory diversification, resulting in functional specialization across the four Mce operons. Among them, Mce1 and Mce4 are currently the best-characterized systems implicated in host lipid uptake, whereas the physiological roles of Mce2 and Mce3 remain less defined.

Taken together, although structural conservation suggests shared mechanistic principles of lipid binding and transport, functional equivalence across systems should not be assumed. Overreliance on cross-system analogy or broad evolutionary inference risks obscuring the pathogen-specific adaptations that define Mce transporter biology in Mtb.

## TRANSLATIONAL IMPLICATIONS OF Mce BIOLOGY

### Diagnostic potential

Conventional tuberculosis diagnostics rely on culture, nucleic acid amplification, or antigen tests, each with practical limitations in speed, sensitivity, or specificity (71–73). Because several Mce proteins are immunogenic and some Mce-regulated lipids appear to differ between wild-type and transporter-defective strains, Mce biology has been explored as a source of candidate diagnostic markers (47, 73–76).

Several Mce proteins, including Mce3C, Mce2D, and Mce3E, have been reported to elicit detectable antibody responses in active tuberculosis, and epitope mapping has identified immunoreactive regions within additional Mce proteins (55–57). These findings support continued exploration of selected Mce antigens as candidate serodiagnostic components.

Beyond protein antigens, nonpolar cell-wall lipids influenced by the Mce1 operon have also shown diagnostic interest in exploratory studies. However, these observations remain early and do not yet establish clinical-grade specificity or reproducibility.

Taken together, available data support further evaluation of selected Mce antigens and Mce-regulated lipids as candidate serodiagnostic components, particularly in combination formats. Substantial validation in larger and more diverse cohorts will be required before stronger claims can be justified.

### Vaccine relevance

Mce systems have also attracted attention in vaccine research because of their contributions to virulence, their immunogenicity, and their absence from the human genome (49, 77–80). Both attenuated mutants and recombinant Mce-derived antigens have shown promise in preclinical settings.

Disruption of individual Mce operons can attenuate Mtb and alter protective immune responses in animal models (77, 78). These findings suggest that Mce-linked pathways can influence vaccine-relevant phenotypes, although the results are not yet sufficiently uniform to define a clear translational ranking among all Mce components.

Recombinant Mce antigens and epitope-based approaches have likewise shown immunogenicity in experimental systems. However, immunogenicity should not be conflated with protective efficacy, and evidence remains largely preclinical and heterogeneous.

Recent TnSeq data further support the view that Mce1-linked functions become important under vaccine-induced immune pressure (80). Overall, current evidence supports continued evaluation of selected Mce-related antigens or attenuated mutants in rational vaccine design, but it does not yet establish Mce-based strategies as validated successors to BCG.

### Drug-target potential

Mce systems are conceptually attractive but still incompletely validated anti-TB targets because they support lipid scavenging, cell-envelope homeostasis, intracellular survival, and virulence, while lacking close human homologs.

The strongest rationale comes from transporter-centered biology. Mce4 functions as a major cholesterol-import pathway and contributes to long-term persistence during infection, whereas Mce1 supports fatty-acid uptake, envelope homeostasis, and adaptation to lipid-rich intracellular environments. These findings suggest that perturbing Mce-dependent lipid acquisition could compromise mycobacterial fitness, particularly under host conditions in which cholesterol and fatty acids become important carbon and energy sources (81, 82).

Recent structural characterization of the Mce1 complex provides a framework for identifying inhibitory sites within the portal, hydrophobic tunnel, Mce-domain ring, YrbE permease module, and ATPase interface. The shared ATPase MceG is also an attractive intervention node because it stabilizes and energizes multiple Mce transporters (9). Regulatory or accessory factors, including Mce1N, LucA, Mce1R, and Mce3R, may provide additional entry points for chemical or genetic perturbation (7, 8, 25, 83). Nevertheless, these possibilities remain largely exploratory: no Mce-targeting compound has yet been established as a validated clinical lead, and future work will need to define druggability, selectivity, resistance potential, and *in vivo* efficacy.

In summary, Mce biology provides several conceptually attractive and pathogen-specific intervention nodes, but the field remains at an early stage from the standpoint of validated therapeutic development. The most realistic near-term value of Mce biology may be to reveal vulnerabilities in lipid uptake and envelope maintenance that can be exploited in combination with existing or emerging anti-TB therapies.

### Conclusions

Several major questions should define the next phase of Mce research in Mtb. The first concerns substrate specificity. Broad assignments such as “fatty acids” and “cholesterol” have been valuable, but they remain incomplete. It will be important to determine whether individual Mce systems distinguish among chain lengths, sterol derivatives, lipid classes, or infection-stage-specific host substrates.

The second concerns native structural biology. Existing structural advances have been transformative, yet higher-confidence models of transporter stoichiometry, conformational cycling, and substrate passage through the multilayered mycobacterial envelope are still needed. This is particularly important for the less well-characterized operons.

The third concerns the roles of accessory and regulatory proteins. Future work should clarify whether Mam, Omam, LucA, MceG, and Mce1N influence assembly, stability, spatial organization, substrate choice, or multiple aspects of transporter function simultaneously. It will also be important to determine whether inhibitory nodes such as Mce1N are counterbalanced by additional positive regulators under specific infection states.

A fourth priority is integration with the larger metabolic network. Lipid transport does not occur in isolation; it intersects with cholesterol catabolism, fatty-acid metabolism, stress adaptation, and infection-stage-dependent gene regulation. The most informative future studies will therefore be those that connect Mce transport activity to the broader physiological programs that sustain persistence in the host.

Finally, translational work will need stronger mechanistic foundations. Progress in diagnostics, vaccine development, or antimicrobial discovery will depend on careful prioritization of the most biologically relevant and experimentally validated Mce functions rather than on broad extrapolation from immunogenicity or operon-level phenotypes alone.

## Data Availability

The original contributions presented in this study are included in the article; further inquiries can be directed to the corresponding authors.
